# Automated AI-based segmentation of canine hepatic focal lesions from CT studies

**DOI:** 10.3389/fvets.2025.1638142

**Published:** 2025-08-06

**Authors:** Silvia Burti, Artur Jurgas, Caterina Puccinelli, Giunio Bruto Cherubini, Simonetta Citi, Alessandro Zotti, Marek Wodziniski, Rachele Brusco, Emma Quaresima, Martina Giordano, Margherita Bendazzoli, Diane Wilson, Nicolò Mastromattei, Tommaso Banzato

**Affiliations:** ^1^Department of Animal Medicine, Production and Health, University of Padua, Padua, Italy; ^2^Department of Measurement and Electronics, AGH University of Krakow, Kraków, Poland; ^3^Department of Veterinary Sciences, University of Pisa, Pisa, Italy; ^4^Information Systems Institute, University of Applied Sciences–Western Switzerland (HES-SO Valais), Sierre, Switzerland; ^5^Antech Imaging Services, Fountain Valley, CA, United States

**Keywords:** liver mass, computed tomography, artificial intelligence, segmentation, dice score, Hausdroff distance

## Abstract

**Introduction:**

Hepatic masses are a common occurrence in veterinary medicine, with treatment options largely dependent on the nature and location of the mass. The gold standard treatment involves surgical removal of the mass, often followed by chemotherapy if necessary. However, in cases where mass removal is not feasible, chemotherapy becomes the primary treatment option. Accurate lesion segmentation is crucial in such scenarios to ensure precise treatment planning.

**Methods:**

This study aimed to develop and evaluate a deep learning-based algorithm for the automatic segmentation of hepatic masses in dogs. To achieve this, 200 canine CT cases with hepatic masses were collected from two clinics and the Antech Imaging Solutions database. Experienced veterinarians manually segmented the lesions to provide ground truth data. 25/200 CTs were excluded because they did not met the inclusion criteria. Finally, the algorithm was built using the nnUNet v2 framework and trained on 130 cases with a 5-fold training scheme. It was subsequently tested on 45 cases.

**Results:**

The algorithm demonstrated high accuracy, achieving an average Dice score of 0.86 and an Average Symmetric Surface Distance (ASSD) of 2.70 mm.

**Conclusions:**

This represents the first report of a deep learning-based algorithm for the automatic segmentation of hepatic masses in dogs using CT imaging, highlighting its potential utility in clinical practice for improved treatment planning.

## 1 Introduction

Hepatic neoplasm is a major health concern in veterinary medicine, and the prevalence of hepatic lesions in neoplasm-affected patients is high (1). The reported incidence of primary hepatic neoplasms ranges from 0.6% to 2.6%. However, most hepatic neoplasms are secondary, with up to 36% of dogs with non-hepatic tumors showing hepatic involvement (1). Surgery is the preferred treatment for primary hepatic neoplasms. However, in some cases, it may not be possible due to factors such as comorbidities, tumor location, and invasion of vascular structures. Recently, stereotactic body radiation therapy has been suggested as an alternative treatment for certain patients with primary hepatic neoplasms (2).

In human medicine, segmenting both the “normal” liver and the hepatic mass is a standard practice, especially in cases where liver resection is the chosen treatment approach. This practice provides essential information about the residual liver volume, which is crucial for surgical planning and postoperative outcomes. Residual liver volume is a key factor in predicting liver function post-surgery, as the liver must retain enough volume and healthy tissue to sustain metabolic demands following resection. Studies have shown that inadequate residual liver volume is associated with higher risks of postoperative liver failure, hence the emphasis on precise volume measurements of both the healthy liver and the mass (3). In veterinary medicine, however, similar standards for assessing residual liver volume post-resection are not well established. Although automatic liver volume estimation methods have been developed for dogs with normal hepatic function (4), these methods primarily focus on assessing the total liver volume in cases of normal liver anatomy. They have not been adapted for segmenting and estimating liver volumes in cases where hepatic masses are present, which would be necessary for determining residual volume following a resection. The lack of standardized guidelines and validated protocols for residual volume assessment in veterinary species limits the application of these techniques in clinical veterinary practice.

Broadly speaking, the applications of artificial intelligence (AI) in veterinary diagnostic imaging can be categorized into three areas: lesion detection, lesion classification, and lesion segmentation (5). The most widely investigated application is, by far, lesion detection, especially on thoracic radiographs (5–9), and orthopedics (10, 11). AI algorithms have been applied for the purpose of classifying lesions across various domains. Notably, they have been utilized in assessing the quality of thoracic radiographic images (12, 13), as well as in forecasting the malignancy of meningiomas (14–16). Semantic segmentation algorithms for automatic lesion segmentation have been developed for the automatic segmentation of retropharyngeal lymph nodes (17), of head and neck tumors (18) and normal kidneys (19).

Lesion segmentation (LS) is a fundamental, yet time-consuming, task in oncological clinical practice since treatment planning is strictly related to dimension and location (18). Furthermore, LS is paramount for assessing the response to treatment in longitudinal imaging studies. Nowadays, LS is often performed manually by well-trained personnel using dedicated software. Manual LS is usually very accurate, but is, as previously mentioned, time-consuming and operator-dependent, and, therefore, AI-based segmentation methods are often preferred over manual LS (3). As far as the authors are aware, no widely available AI-driven software exists for the automatic segmentation of CT or MR images. However, in human medicine, several pieces of software have received FDA clearance (https://www.fda.gov/medical-devices/software-medical-device-samd/artificial-intelligence-and-machine-learning-aiml-enabled-medical-devices). This study aims to develop and test an AI-based algorithm for automatically segmenting liver masses from canine CT studies.

## 2 Materials and methods

### 2.1 Data collection

The primary goal of this data collection was to build a comprehensive dataset of CT studies featuring hepatic masses in dogs. This dataset would be utilized to train, test, and validate an automatic segmentation algorithm. To enhance the algorithm's generalizability and reduce the risk of selection bias, we aimed to include data from the widest possible range of CT scanners. To gather the data set, we retrospectively searched the databases of the University of Padua, the Pedrani Clinic, and Antech Imaging Services for CT studies conducted in dogs with liver masses or nodules between January 2019 and December 2023. Ethical approval was waived due to the retrospective nature of the study. Informed consent for personal data processing was obtained from the owners. All experiments were performed in accordance with relevant guidelines and regulations. Keywords, such as “Hepatic Mass,” “Liver Mass,” “Liver nodule,” and “HCC,” were employed to retrieve relevant studies. To date, there is no widely accepted distinction between nodules and masses. Therefore, both terms were used in the search. Due to the broad nature of these search terms, the initial results included many unrelated cases, which were then manually filtered based on CT reports to identify the relevant studies. The selected cases were saved in Digital Imaging and Communications in Medicine (DICOM) format in a separate folder, ensuring that both the CT scans and associated reports were fully anonymized. Only delayed-phase CT scans were used for lesion segmentation. As stated above, due to the lack of a widely accepted distinction between nodules and masses, we included all CT scans with up to two focal liver lesions in the initial phase CT scans showing disseminated liver disease were excluded.

### 2.2 Ground truth determination

To train segmentation algorithms, it is essential to create segmentation masks, that are binary volumes that capture the spatial information of the lesion within the selected volume. To generate these masks, we used the free software 3D Slicer (20). The selected volumes were imported into 3D Slicer and visually inspected to ensure the absence of significant imaging artifacts and to assess image quality.

The contouring process began with manual delineation of the lesions on a subset of images. Gaps were then filled using pre-installed automatic filling tools. However, because lesions often have irregular shapes, the automatic tools frequently fail to produce accurate contours, necessitating manual adjustments. These steps are crucial since the lesions usually span multiple CT slices. Manually contouring every slice, particularly in cases of large lesions that can extend across hundreds of contiguous slices, would be extremely time-consuming. Finally, the original volume and the segmentation mask were saved in different files.

In the current study, we opted not to segment the normal liver due to the absence of specific veterinary guidelines on residual liver volume assessment. Without established thresholds or criteria for “safe” residual liver volume in dogs, segmenting only the mass provides sufficient information for diagnostic purposes without the added complexity of delineating normal hepatic tissue.

### 2.3 Model architecture

In this study, we employed the nnUNet v2 framework, widely regarded as a state-of-the-art solution for medical image segmentation tasks (21). This framework offers a robust baseline and supports extensive experimentation due to its customizable architecture. The nnUNet v2 model is built on the U-Net architecture, featuring an encoder-decoder structure with both short and long skip connections (22). The encoder extracts image features by progressively downsampling the input, while the decoder generates the output segmentation mask by upsampling from the latent feature space. For this study, we used the Residual Encoder (ResEnc) variant (23) of nnUNet v2 in size L. This variant integrates residual connections within the encoder to mitigate the vanishing gradient problem during training, allowing for the use of deeper networks. Residual connections also enhance information flow across layers, contributing to more efficient learning.

As optimal configurations for nnUNet are currently undetermined, we trained four distinct configurations to evaluate performance.

1. **2D model:** This model worked by processing individual slices of the volumetric CT scans, treating each 2D slice independently. This approach is computationally efficient and is particularly useful when the inter-slice context is less critical for the segmentation task.
2. **3D low-resolution model:** This model used downsampled 3D volumes, allowing it to capture spatial context across multiple slices. This configuration balances the use of 3D context while reducing memory requirements during training.
3. **3D full-resolution model:** This model operated on high-resolution 3D data, preserving the full detail of the original scan. It leverages detailed spatial relationships in the volume, though it is more computationally demanding.
4. **3D cascade model:** This approach combined both the 3D low-resolution and 3D full-resolution models. It first used the 3D low-resolution model to generate a rough segmentation, then refined it using the 3D full-resolution model.

#### 2.3.1 Training configuration

**Figure 1:**
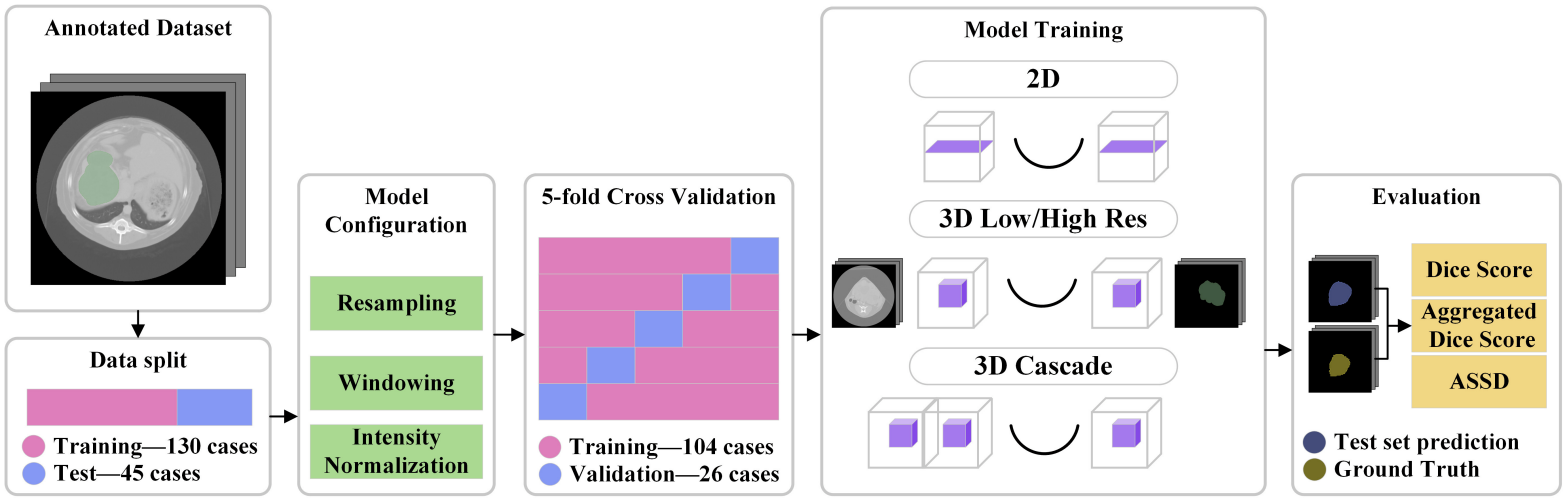
Pipeline visualization. A separate test set of 45 cases was split from the annotated data. All the training files influenced the model configuration. The training was done on 5 folds of the training data. Four different models were trained, and finally the test data was used for evaluation.

The model was trained from scratch on the dataset using a 5-fold cross-validation approach. The data was split into training and validation sets in the 80/20 ratio for each of the folds. A separate set of 45 cases was reserved for testing, allowing us to evaluate the generalization capability of the trained model. The training and evaluation pipeline is visible in Figure 1.

For each fold, we trained a separate model with the specified configuration. This means that for each model in Table 1, five individual models were trained, one for each fold. During inference, all five models were used, combining their predictions by summing the logits (the model's output values before applying the sigmoid function) and then averaging the sum by dividing it by 5, the number of folds.

**Table 1:** Preprocessing configuration for each of the models.

**Model**	**Spacing**	**Patch size**	**Batch size**
2D	0.586, 0.586	512, 512	35
3D low-resolution	1.583, 0.811, 0.811	128, 256, 224	2
3D full-resolution	1.250, 0.586, 0.586	112, 224, 224	2
3D cascading	3D Low-Res → 3D Full-Res		

The training parameters, including learning rates and batch sizes, were automatically adapted based on the characteristics of the data. Details of the training setup are provided in Table 1. The input data consisted of CT images of liver lesions. These images were preprocessed by resampling to a common voxel spacing of 1 mm and then normalized.

#### 2.3.2 Loss function and optimization

We used a Soft Dice Coefficient combined with Cross-Entropy loss as the objective function during training. For optimization, the Stochastic Gradient Descent (SGD) optimizer was employed, with an initial learning rate of 1 × 10^−2^, weight decay of 3 × 10^−5^, and Nesterov momentum of 0.99. A polynomial learning rate decay schedule was used to adjust the learning rate over the course of training.

The model was trained using a 5-fold cross-validation approach. The training data was split into training and validation sets with the 80/20 proportion for each of the folds. Additional X cases were used as a test set to evaluate the generalization capability of the model.

All training was done on 12 CPU cores configured with 15 Gb of memory for each. For GPU, an Nvidia A100-SXM4-40GB model was utilized with 400 W of power draw with CUDA version 12.1.

### 2.4 Evaluation metrics

Evaluating automatic segmentation methods involves using some metrics that might be unfamiliar to some readers. Indeed, a pixel-wise comparison of the correspondence between the ground truth for the segmentation performed by the experts and the segmentation predicted by the algorithm has to be performed. To this end, three fundamental metrics are used: the Dice similarity coefficient (DSC), Aggregated Dice Similarity Coefficient (DSCAgg), and the Average Symmetric Surface Distance (ASSD).

The DSC is calculated using the following formula:


(1) $$\begin{array}{l} DSC\;=\frac{2\left(A\cap B\right)}{\left(\left|A|+|B\right|\right)} \end{array}$$

Basically, the Dice similarity coefficient is a measure used to calculate how similar two sets or groups are—the ground truth for the segmentation performed by the experts and the segmentation predicted by the algorithm.

In addition to the DSC, the Aggregated Dice Similarity Coefficient (DSCAgg) is also considered in this analysis. Unlike the traditional DSC, which calculates the Dice score for each individual case and then takes the mean, DSCAgg accumulates all True Positives (TP), False Negatives (FN), and False Positives (FP) across all cases before calculating the Dice score. This approach minimizes the disproportionate influence that smaller lesions can have on the average Dice score in the traditional calculation, where they may excessively impact the overall result.

The DSCAgg is computed as follows:


(2) $$\begin{array}{l} DSC_{Agg}=\frac{2\times TP}{2\times TP+FN+FP} \end{array}$$

This aggregated approach offers a more balanced assessment of segmentation accuracy across cases of varying lesion sizes.

The Average Symmetric Surface Distance (ASSD) is calculated using the formula:


(3) $$\begin{array}{l} ASSD=\frac{1}{\left|A|+|B\right|}\left(\underset{a\in A}{\sum }\underset{b\in B}{\inf}d\left(a,b\right)+\underset{b\in B}{\sum }\underset{a\in A}{\inf}d\left(b,a\right)\right) \end{array}$$

The Average Symmetric Surface Distance is a measure of the average distance between the surfaces of two shapes, A and B. It calculates the mean of the minimal distances from each point on the surface of one shape to the nearest point on the surface of the other shape, considering both directions (from A to B and from B to A). This metric provides a balanced average rather than capturing the maximum distance, making it useful for assessing the overall similarity between two shapes.

## 3 Results

### 3.1 Dataset

The initial database query retrieved an excessively large number of studies (over 10,000), primarily due to the lack of specificity in the keywords used. From the retrieved studies, 200 CT studies with focal liver lesions were selected for training and testing the algorithm. Since there is no widely accepted size threshold to differentiate between a mass and a nodule (e.g., a 1 cm lesion may be considered small in a Great Dane but large in a Toy Poodle), we opted to include all CT studies with maximum two hepatic focal lesions, regardless of size. A total of 25 out of 200 CT scans were excluded after manual inspection revealed the presence of more than two lesions, leading to their exclusion. The average size of the lesions used in the study was 75.35 × 63.64 × 66.94 mm. Out of the 175 available scans, 130 were randomly chosen to be part of the training set, with the remaining 45 allocated to the test set.

The CT studies used in this work were acquired using a multitude of CT scanners, and, as a result, the acquisition parameters had quite a large variability both in terms of slice thickness and of radiological parameters. CTs were acquired with the following CT scanners (GE MEDICAL SYSTEMS Brivo CT385 Series, GE MEDICAL SYSTEMS LightSpeed Plus, GE MEDICAL SYSTEMS LightSpeed Pro 32, GE MEDICAL SYSTEMS LightSpeed Ultra, GE MEDICAL SYSTEMS LightSpeed VCT, GE MEDICAL SYSTEMS LightSpeed 16, GE MEDICAL SYSTEMS Revolution ACT, International Medical Solutions, Neurologica CereTom, SIEMENS Emotion 16, SIEMENS Spirit, TOSHIBA Aquilion, TOSHIBA Aquilion Lightning).

The manual segmentation process took between 10 and 40 min per case, though the exact time was not systematically recorded. The duration varied based on the size and CT characteristics of the lesions. In cases where the lesion was clearly distinguishable from the normal liver parenchyma, automatic filling tools were able to reliably complete the segmentation between the manually outlined slices. However, when the contrast between the lesion and the surrounding liver tissue was less distinct, the automatic tools were less effective, requiring the operator to spend additional time refining and adjusting the automatically segmented regions.

### 3.2 Model performance

**Figure 2:**
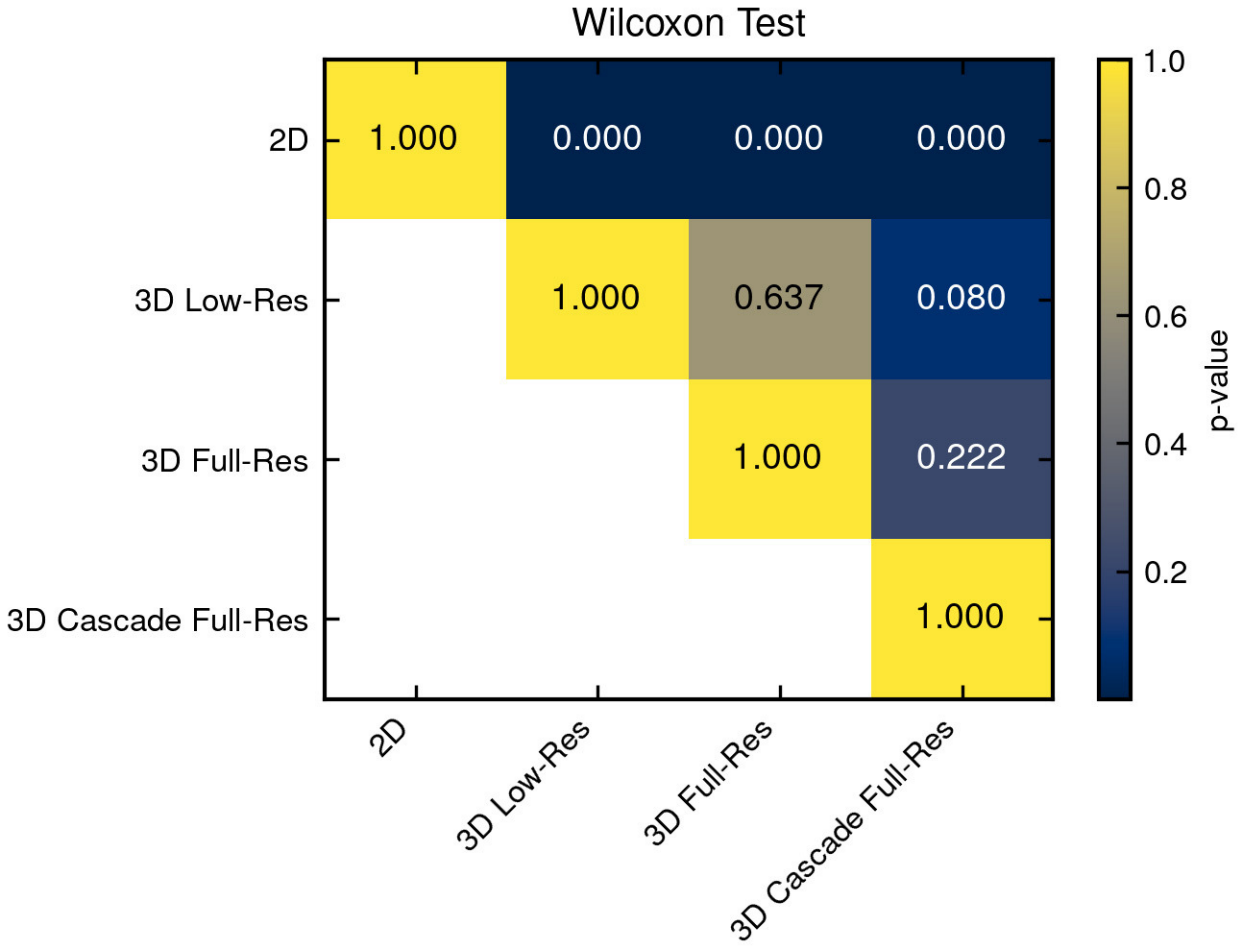
Statistical significance among all compared models was assessed. Differences from the 3D models were not statistically significant, whereas the 2D model exhibited significantly inferior performance.

Model performance was calculated only on the 45-case test set. The performance of each model is summarized in Table 2, showcasing key metrics for Dice score, aggregated DSC, ASSD, and training time. The 3D high-resolution cascade model achieved the highest overall Dice score (0.86) and lowest ASSD (2.70 mm), demonstrating superior segmentation quality, though it required a significantly longer training time of 115 h. The 3D low-resolution model ranked as the second-best performer, achieving a Dice score of 0.85 and an ASSD of 2.77 mm, closely matching the cascade model while maintaining a shorter training time of 33 h. While the 3D full-resolution model operates at a higher resolution, the 3D low-resolution model benefits from a larger receptive field, enabling it to capture broader spatial context, which appears advantageous in lesion segmentation. It's important to highlight that, despite the variance in average performance, the statistical analysis presented in Figure 2 did not reveal any significant differences among the 3D models. In this context, the 3D Low-Res model might be the optimal choice for practical clinical applications, especially when time is a limiting factor.

**Table 2:** Model performance comparison for liver lesion segmentation.

**Model**	**Dice**	**DSC (Agg)**	**ASSD [mm]**	**Training time [h]**
2D	0.68	0.80	6.16	28
*3D low-resolution*	*0.85*	*0.93*	*2.77*	*33*
3D full-resolution	0.82	0.92	3.54	42
**3D cascade**	**0.86**	**0.93**	**2.70**	**115**

Best results have been presented in bold, with the second-best in italic.

In contrast, the 2D model exhibited the lowest Dice score (0.68) and the highest ASSD (6.16 mm), reflecting its limitations in capturing 3D spatial context necessary for accurate lesion delineation. The 2D nnU-Net analyzes each individual image slice separately, without considering the anatomical structures present in adjacent slices. In veterinary imaging, where the contrast between soft tissues is often poor, this break in spatial continuity probably hindered the model's precision in delineating structures. This is further supported by the statistical significance analysis presented in Figure 2, where a Wilcoxon signed-rank test reveals that the performance of this model is significantly distinct from that of the 3D models.

The model's ability to avoid delivering false positives was assessed using 72 cases without obvious liver masses. The 2D and 3D Low-Resolution models incorrectly identified liver masses in only 2 instances (2.78%), while the 3D Full-Resolution model resulted in 3 inaccurate predictions (4.17%). The 3D Cascading model yielded 4 false positives among the 72 cases (5.56%).

**Figure 3:**
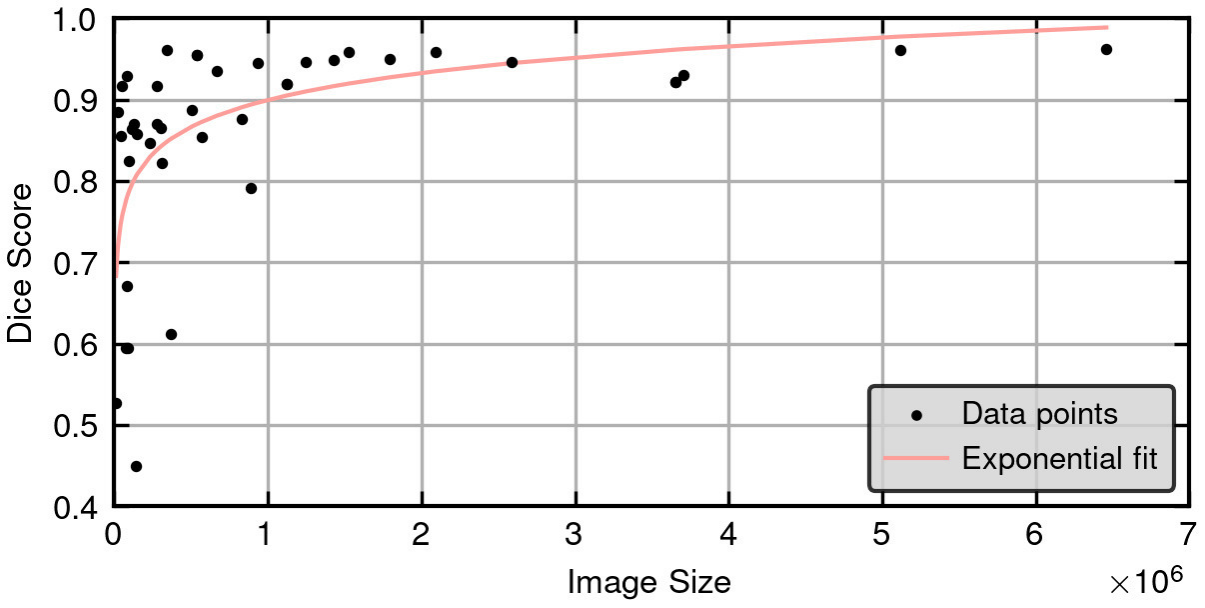
Scatter plot illustrating the relationship between Dice scores and ground truth lesion size for the 3D high resolution cascade model in the test set (45 cases), showing a clear positive correlation between lesion size and segmentation accuracy.

**Figure 4:**
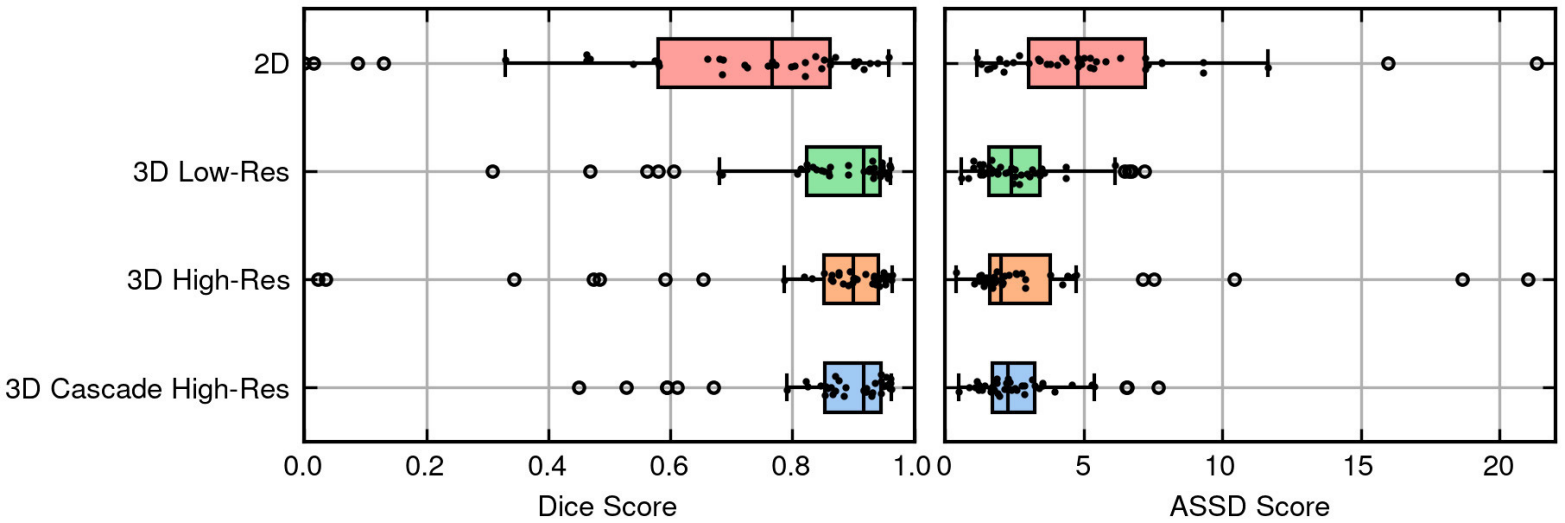
Box and swarm plots merged to display the performance metrics of models, with each dot representing a single observation.

Figure 3 provides a scatter plot illustrating the relationship between Dice score and lesion size for the 3D cascade model. Notably, Dice scores improve with increasing lesion sizes according to a positive regression coefficient—indicating that segmentation quality may vary based on lesion characteristics. Figure 4 presents a plot comparing the spread of Dice and ASSD scores across all models, highlighting that the 3D cascade and 3D low-resolution models show less variability, while the 2D model displays a wider range, particularly in ASSD values.

**Figure 5:**
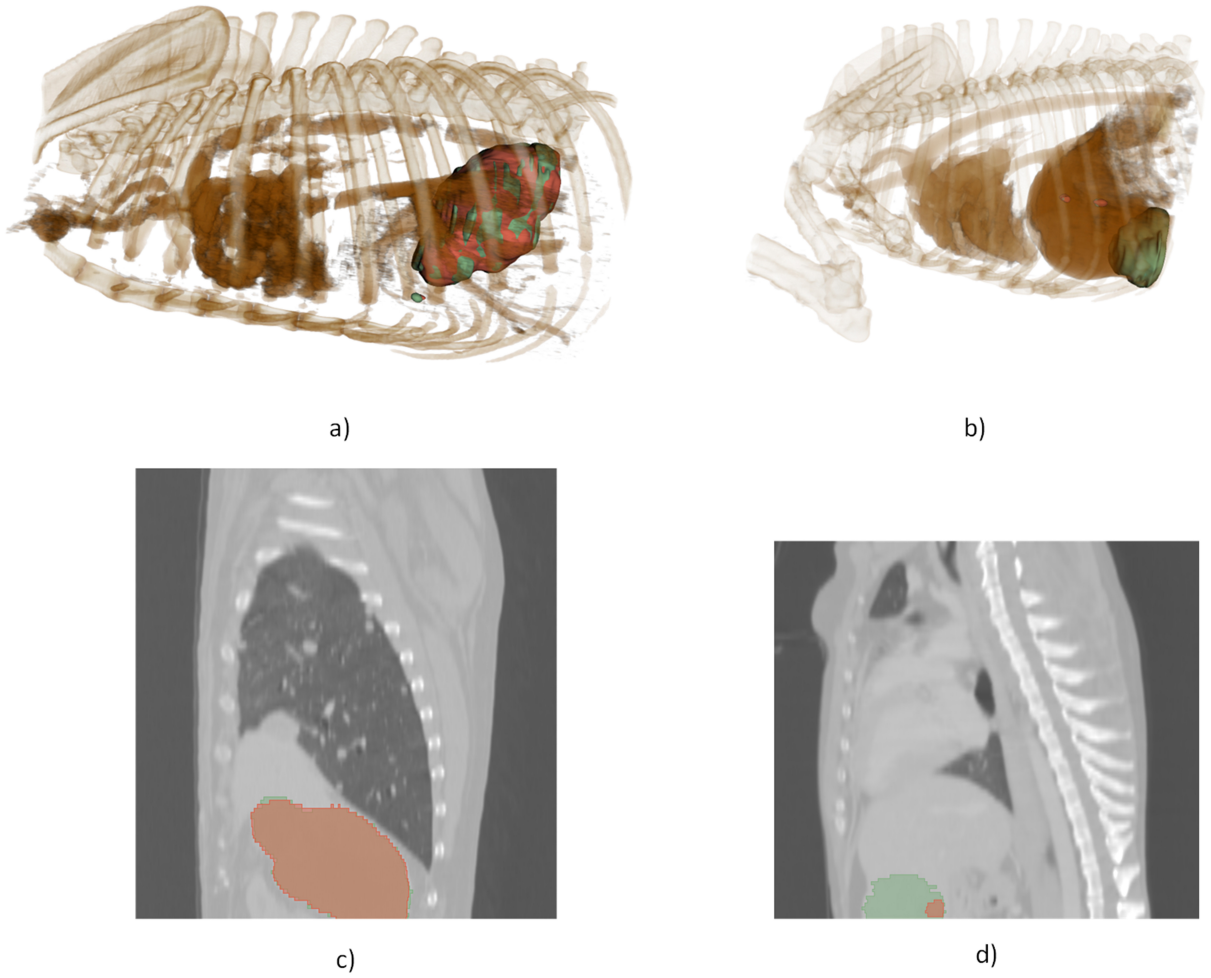
3D visualization, illustrating a comparison between the model's most accurate and least accurate segmentations. **(a)** 3D rendering of the optimal prediction, **(b)** 3D rendering of the poorest prediction, **(c)** 2D slice from the optimal prediction, **(d)** 2D slice from the poorest prediction.

Finally, Figure 5 offers a qualitative visualization, demonstrating examples of the best and worst segmentation outcomes from the 3D cascade model using 3D Slicer. This visual inspection underscores the high fidelity of the model's best-case predictions and highlights areas for improvement in challenging cases. Together, these figures emphasize the impact of model architecture on segmentation performance, particularly for complex lesion structures.

## 4 Discussion

This is the first study to describe the development of a deep learning-based tool for automatic segmentation of liver masses in veterinary medicine. The algorithms developed demonstrated robust performance, achieving an overall Dice score of 0.86 and an aggregated Dice of 0.93 (with the Dice score ranging from 0 to 1) in the test set. Such performance is especially noteworthy given the diverse range of scanners and settings used to acquire the scans for training and developing the models. Indeed, all previous studies on segmentation tools in veterinary medicine are monocentric retrospective studies (18, 24), meaning that the generalization ability of the developed networks has not yet been extensively tested. It should be noted that while the model's accuracy has been confirmed with this particular test set composed of 45 cases, its “real-world” accuracy is yet to be determined.

Segmentation is used primarily in oncological settings to assess the overall dimensions of a mass and to precisely delineate the contours of the lesion, especially in cases where non-invasive treatments such as radiofrequency ablation are considered (3). Currently, there are several segmentation methods available and no one approach fits all clinical needs (25). Manual segmentation is becoming less common in human medicine as powerful automatic or semi-automatic segmentation tools are increasingly accessible (26). The workflow proposed in this study is fully automated, but once implemented in a convenient tool with the possibility of correcting the automatically proposed segmentations, a semi-automated method can be implemented.

In the current study, we opted not to segment the normal liver due to the absence of specific veterinary guidelines on residual liver volume assessment. Without established thresholds or criteria for “safe” residual liver volume in dogs, segmenting only the mass provides sufficient information for diagnostic purposes without the added complexity of delineating normal hepatic tissue. However, the development of standardized guidelines in veterinary medicine for post-resection liver volume assessment could significantly enhance surgical planning and postoperative care in the future.

AI-based automatic segmentation of CT images is still seldom explored in veterinary medicine, limiting direct comparisons between our results and existing veterinary literature. However, the human medical literature on automatic segmentation of hepatic masses is more comprehensive. A recent review by Wesdorp et al. (27) on AI-based liver segmentation methods reports Dice scores (DS) ranging from 0.569 (28) to 0.940 (29) across various human studies. Most studies in human medicine utilize the LiTS17 (Liver Tumor Segmentation Challenge 2017) dataset, which consists of 201 segmented CTs (131 for training and 70 for testing) (25). In this regard, our dataset is comparable in size, and the results obtained align with those of top-performing algorithms in human medicine.

In the last few years some papers describing the potential use of U-Net and its different versions for segmentation tasks in veterinary diagnostic imaging were published. Groendahl et al. (24) trained a UNet in dogs with spontaneous head-and-neck cancer that, after pre-training on human cases and fine-tuning on 36 canine CT studies, reached median Dice scores around 0.56 and patient-level values up to 0.89 for gross-tumor-volume delineation. The same architecture was also applied to much smaller structures: Schmid et al. (17) used a 2-D U-Net to segment medial retropharyngeal lymph nodes using only 40 canine heads as training and test set; despite the limited data, the network obtained a median intersection-over-union of 0.39. Ji et al. (19) combined U-Net with transformer blocks and trained it on 182,974 CT slices from 211 dogs, achieving a Dice coefficient of 0.92 ± 0.05 for renal parenchyma and enabling fast kidney-volume reference curves useful in clinical nephrology. Finally, Park et al. (30) developed a UNet for the automatic calculation of adrenal gland volume from CT images achieving a Dice score of 0.885 ± 0.075 and therefore allowing fast and precise estimation of adrenal volume.

One notable limitation identified in this study is the relationship between the accuracy of the model and the size of the lesion. As illustrated in Figure 3, there is a clear and statistically significant correlation between lesion size and the Dice similarity coefficient, suggesting that the performance of the segmentation of the model varies with the dimensions of the lesion. Specifically, larger lesions tend to yield higher Dice scores, indicating more accurate segmentation, while smaller lesions exhibit lower scores. From a clinical standpoint, this correlation may present both challenges and advantages. On the one hand, accurately segmenting smaller lesions remains a critical but demanding task, potentially requiring more sophisticated modeling techniques to achieve higher precision. On the other hand, the model's enhanced accuracy with larger lesions can be advantageous, as segmenting extensive lesions is typically more time-consuming and labor-intensive for clinicians. By automating the segmentation of larger lesions with greater accuracy, the model can significantly reduce the manual workload and expedite the diagnostic process. Moreover, these findings are consistent with observations in human studies. For instance, Li et al. (31) reported similar trends, although their study did not explicitly quantify the correlation between lesion size and segmentation accuracy. This alignment with existing research underscores the validity of our results and highlights the model's potential applicability in clinical settings. However, the absence of reported correlations in human studies also points to an area for future research, where further investigation could elucidate the underlying mechanisms driving the relationship between lesion size and segmentation performance.

## 5 Conclusion

In the present study, we propose a fully automatic segmentation method for hepatic focal lesions in canine CT images. The best-performing model (3D high resolution cascade model) demonstrated a high level of accuracy, with a Dice Similarity Coefficient (DSC) of 0.86 and an Average Symmetric Surface Distance (ASSD) of 2.7 mm. These metrics indicate strong model performance, as the DSC of 0.86 reflects excellent overlap between the predicted and actual lesion boundaries, and the ASSD of 2.7 mm suggests minimal surface deviation, aligning well with clinical expectations. Notably, these results are comparable to the state-of-the-art segmentation models currently in use in human medicine, where automated liver lesion segmentation has achieved high precision.

However, while these initial results are promising, further improvements could be achieved by expanding the training dataset. Increasing the dataset size would likely enhance the model's robustness and generalizability across various lesion presentations, ultimately improving accuracy. A larger dataset could also capture a broader range of hepatic lesion morphologies, sizes, and contrast characteristics, which would allow the model to perform well even in cases with less typical lesion appearances. This expansion could be particularly valuable in addressing any potential model biases introduced by a limited sample size, thereby enhancing the model's clinical utility across diverse cases.

## Data Availability

The datasets presented in this article are not readily available because of GDPR regulations. Requests to access the datasets should be directed to tommaso.banzato@unipd.it.
